# EASe-COVID: Evaluation of Anxiety and Solitude in COVID-19 wards

**DOI:** 10.1192/j.eurpsy.2023.1699

**Published:** 2023-07-19

**Authors:** N. Waheed

**Affiliations:** Psychiatry, Herefordshire and Worcestershire Healthcare and care NHS trust, Worcester, United Kingdom

## Abstract

**Introduction:**

COVID-19 has had a significant impact on our daily lives in a variety of ways. In hospital settings, patients who are admitted on COVID-19 wards are usually isolated from their family and friends. This, in turn, can lead to patients feeling lonely and having increased level of anxiety.

**Objectives:**

To assess the level of anxiety and feeling of loneliness amongst the patients during their in-patient stay in a COVID-19 ward.

We aimed to highlight what could be done differently to reduce the amount of anxiety amongst the patients.

**Methods:**

We carried out EASe-COVID study to assess level of anxiety and feeling of loneliness amongst the patients during their in-patient stay in a COVID-19 specific ward. We designed 2 questionnaires – 1 for patients, using the GAD-7 anxiety questionnaire and UCLA 3-item loneliness scale and an open-ended questionnaire for staff members. Questionnaires were distributed from January – March 2022 to randomly selected members of healthcare staff and patients on COVID wards. 15 patients returned the anonymised questionnaire, whereas 11 staff members returned the completed questionnaire.

**Results:**

Most of the patients were satisfied with the patient care they received during their stay and did not feel increasingly anxious or lonely during their inpatient stay in the COVID wards. On the other hand, members of the healthcare team felt that they were short staffed and under-trained to deal with the complex patients on the COVID ward. Not being able to spend enough time reassuring patients was a common theme in the responses from the staff questionnaires.

**Conclusions:**

The in-patient stay on COVID wards was generally a positive experience for the patients. However, the study highlighted that the visitation rules and the short staffing were the main issues contributing to anxiety and loneliness highlighted by both staff and patients. It was clear that the staff had a patient centred approach to care, but felt limited by time, experience and staffing.

**Disclosure of Interest:**

None Declared

